# Rational Design of a User-Friendly Aptamer/Peptide-Based Device for the Detection of *Staphylococcus aureus*

**DOI:** 10.3390/s20174977

**Published:** 2020-09-02

**Authors:** Luca Ronda, Alessandro Tonelli, Elisa Sogne, Ida Autiero, Francesca Spyrakis, Sara Pellegrino, Giorgio Abbiati, Elisa Maffioli, Carsten Schulte, Riccardo Piano, Pietro Cozzini, Andrea Mozzarelli, Stefano Bettati, Francesca Clerici, Paolo Milani, Cristina Lenardi, Gabriella Tedeschi, Maria Luisa Gelmi

**Affiliations:** 1 Department of Medicine and Surgery, University of Parma, 43125 Parma, Italy; luca.ronda@unipr.it (L.R.); riccardo.piano@studenti.unipr.it (R.P.); 2 Institute of Biophysics, National Research Council, 56124 Pisa, Italy; andrea.mozzarelli@unipr.it; 3 DNAPhone s.r.l., 43126 Parma, Italy; alessandro.tonelli@dnaphone.it; 4 CIMAINA and Department of Physics, University of Milan, 20133 Milan, Italy; eli.sogne@gmail.com (E.S.); carsten.schulte@unimi.it (C.S.); paolo.milani@mi.infn.it (P.M.); cristina.lenardi@mi.infn.it (C.L.); 5 Molecular Horizon s.r.l, 06084 Perugia, Italy; ida@moldiscovery.com; 6 Institute of Biostructures and Bioimaging, National Research Council, 80145 Naples, Italy; 7 Department of Drug Science and Technology, University of Turin, 10125 Turin, Italy; francesca.spyrakis@unito.it; 8 Department of Pharmaceutical Sciences, University of Milan, 20133 Milan, Italy; sara.pellegrino@unimi.it (S.P.); giorgio.abbiati@unimi.it (G.A.); francesca.clerici@unimi.it (F.C.); marialuisa.gelmi@unimi.it (M.L.G.); 9 CIMAINA and Department of Veterinary Medicine, University of Milan, 20133 Milan, Italy; elisa.maffioli@unimi.it (E.M.); gabriella.tedeschi@unimi.it (G.T.); 10 Department of Food and Drug, University of Parma, 43124 Parma, Italy; pietro.cozzini@unipr.it

**Keywords:** *Staphylococcus aureus*, biosensors, molecular dynamics, circular dichroism, fluorescence, nanostructured surface, point-of-care detection

## Abstract

The urgent need to develop a detection system for *Staphylococcus aureus*, one of the most common causes of infection, is prompting research towards novel approaches and devices, with a particular focus on point-of-care analysis. Biosensors are promising systems to achieve this aim. We coupled the selectivity and affinity of aptamers, short nucleic acids sequences able to recognize specific epitopes on bacterial surface, immobilized at high density on a nanostructured zirconium dioxide surface, with the rational design of specifically interacting fluorescent peptides to assemble an easy-to-use detection device. We show that the displacement of fluorescent peptides upon the competitive binding of *S. aureus* to immobilized aptamers can be detected and quantified through fluorescence loss. This approach could be also applied to the detection of other bacterial species once aptamers interacting with specific antigens will be identified, allowing the development of a platform for easy detection of a pathogen without requiring access to a healthcare environment.

## 1. Introduction

*Staphylococcus aureus*, a common commensal of skin and nares, is one of the most frequent causes of infections [1,2], one of the five most common causes of infections after injury or surgery. Moreover, it has become the second major bacterium in food poisoning since it is able to produce heat-resistant toxins [3]. In addition, antibiotic-resistant strains of *S. aureus* (e.g., methicillin-resistant *staphylococci*) appeared not only in clinical settings such as in hospitals, but also in whole communities [4]. These premises make the development of rapid and reliable methods to identify *S. aureus* in biological samples as well as in food an urgent need to avoid epidemics.

Pathogen detection mainly relies on microbiological and biochemical methods, but these are usually time-consuming, expensive and not suitable for integration in on-site diagnosis. In fact, conventional methods to detect *S. aureus*, such as cell cultures, require more than one day and cannot distinguish among strains belonging to other species such as *S. intermedius*, *S. caprae*, *S. simulans* and *S. capitis* [5]. Rapid *S. aureus* agglutination tests have been developed as an alternative for routine diagnosis, but their accuracy, specificity and predictive capacity have been questioned [6,7,8]. Techniques involving manual or automated biochemical methods exploiting colorimetric reactions are widely used in clinical laboratories. They are faster than cell cultures and comparative studies indicate significantly better results [9], but they require specific instrumentation and trained personnel. For these reasons, there is currently a huge demand for alternative, rapid methods to detect *S. aureus* overcoming these limitations [10,11]. The advent of real-time PCR led to the development of rapid methods (a few hours), not requiring the bacteria isolation [12,13,14], but this technique requires expensive equipment and trained personnel. Hence is not suitable for bedside point-of-care use. Moreover, PCR-based methods are based on nucleic acids amplification, hence suffer from false positive results coming from contaminants sequences amplification, as well as false negative results coming from template nucleic acid degradation. As an alternative to nucleotides, peptide nucleic acids (PNAs), pseudopeptides mimicking DNA, show improved binding behavior and higher stability, but their cost is still too high for their application in this field. MALDI-TOF MS-based identification of bacteria is a fast and accurate technology [15,16,17,18] and could be an alternative to molecular tests if the test accuracy is proven [19], but requires an expensive instrument as well. Beside their interest and popularity, the costs, required time and the need for trained personnel and fixed equipment make the applicability of these techniques to point-of-care pathogen monitoring tools challenging to realize.

An attractive alternative is represented by biosensors, devices where a biological component, such as a protein or oligonucleotide, is coupled with a transducer for obtaining a readable signal. Biosensors may represent novel and user-friendly devices to handle human, animal of food samples, allowing the rapid detection of bacteria without the need for expensive and fixed equipment.

The recognition of a bacterial pathogen such as *S. aureus* has been approached in different ways by biosensors, exploiting proper transducers to give a readable signal (Table 1). Commonly reported recognition tools are: (1) antibodies [20]; (2) bacteriophages [21]; (3) phage display peptides and phage receptor binding proteins and (4) nucleic acids. While successful in immuno-analytic protocols, antibodies are still costly, and show a relatively short shelf life and poor stability towards changes in temperature, pH and ionic strength. Moreover, ethical issues regarding the need for animal immunization for their production have to be considered. However, optical fibers-based biosensors conjugated to monoclonal antibodies that bind methicillin-resistant *Staphylococci* have been recently reported as a potential alternative to cell cultures [22]. Phages, which have better stability, and are selective for single strains of bacteria, have still to be optimized in terms of immobilization density and their purification remains challenging [23]. Nucleic acids are exploited for their capability to complementary hybridize each other or to form more complex tridimensional structures able to selectively recognize epitopes, similarly to antibodies.

As an example of the first case, an electrochemical DNA sensor based on DNA hybridization has been recently developed and tested on contaminated food [24]. The second case is mainly represented by aptamers, a novel and highly performing analytical tool for diagnostic applications [25]. Aptamers are DNA or RNA segments acting as artificial recognition elements that are able to recognize conserved epitopes on the surface of a bacterium. Aptamer libraries are continuously growing thanks to the recent progress in the aptamer selection procedure (SELEX). Moreover, due to the relatively easy prediction of their secondary structure, the possibility to modulate affinity towards a target by a rational modification of the sequence has been demonstrated [26]. While several aptamers are available for recognizing *S. aureus* [27,28,29,30,31,32,33] and hence a set of molecular recognition probes is available, the development of a proper transducer for a biosensor is still calling for an intensive research in the field of the technological challenges in translating aptamer-based biosensors in clinical practice is the possibility to have substrate materials adequate for the high density and functional immobilization of aptamers. A first endeavor to overcome this limit relies in the use of carbon nanotubes (CNTs), chosen for their capability to form hybrids with nucleic acids and transducing electrical signals with high efficiency, exploited in the development of electrochemical sensors [10,34,35]. An ultra-sensitive method for bacterial identification based on the resonance light-scattering signal of aptamer-conjugated gold nanoparticles was used for detecting single *S. aureus* cells [36]. Other recent advances in the development of aptamer-based *S. aureus* biosensors are based on gold electrode piezoelectric sensors [30] or silver nanoparticles [31].

Despite these encouraging results, the development of surfaces able to immobilize aptamers is still crucial and nanostructured surfaces are very promising for this kind of applications [37,38,39,40,41,42], because they can allow high-density immobilization of biorecognition elements while retaining their structure and functionality. In particular, it has been demonstrated that the surface nanoscale morphology promotes protein [43,44,45] and aptamer [46] adsorption. Thus, producing nanostructured substrates with well controlled and scalable roughness represents a unique asset for the implementation of aptamers-based devices.

We took advantage of aptamers already known to interact with *S. aureus* to improve their surface immobilization density, by adopting a nanostructured zirconium dioxide support to promote surface linking. The final aim is to produce a detection system kit suitable for point-of-care use with a user-friendly read-out. We proved the possibility of creating a detection kit by immobilizing on the nanostructured support one of the highest affinity reported aptamers, SA23 [27]. The detection is based on a fluorescein-labeled peptide bound to the aptamer that is displaced in the presence of bacteria and released free in the solution, with a concomitant change of its fluorescence properties. Pathogens can be detected by visual inspection, ideally simply by illuminating the device with a visible commercial blue led.

The novelty of our biosensor development approach resides in coupling an already known biorecognition element obtained with a high throughput approach (i.e., aptamers) with a rationally designed element (specifically interacting peptides), to give an easily readable signal. To design the structure of peptides we introduced in silico design in the development of a transducer [47,48], i.e., a peptide able to selectively bind an aptamer based on its sequence.

The interacting peptide was designed in silico exploiting the energetic amino acid-base recognition code previously obtained by estimating the interaction energy of several protein-DNA complexes with the HINT force field [49]. The latter, originally developed for evaluating protein-ligand interactions [50], has also been successfully applied to protein-protein [51], protein-water [52] and protein-DNA [49,53] systems. Differently from complementary double stranded DNA, aptamers do not have a well-defined three-dimensional structure and present a high level of flexibility [54]. It is known that aptamers are predominantly unstructured molecules in solution, and fold upon association with the corresponding ligands into molecular architectures, in which the ligand becomes an intrinsic part of the nucleic acid structure [55]. This property was favorably exploited to increase the energy of interaction of peptides specifically designed to bind aptamers. Starting from the aptamer sequence, the aforementioned recognition code was used to predict the residues able to give stronger interactions, considering that contacts as Arg and Lys with G, Asp and Glu with C, and Asn and Gln with A have demonstrated to be conserved and to better stabilize protein-nucleic acid complexes [49]. The designed peptides were then synthesized using stepwise solid phase synthesis (SPPS), exploiting the effectiveness of microwave irradiation, which previously demonstrated its potential allowing the synthesis of a 75mer peptide [56].

We finally created a device where the fluorescein-labeled peptide, hybridized to the aptamer immobilized on the nanostructured surface, is displaced by *S. aureus* and released in the biological fluid, losing its fluorescence. The properties of nanostructured materials could also fit in microfluidic devices, that demonstrated to be useful in the concentration of pathogens, hence improving the detection limit [57].

This approach can in principle be expanded towards any biological agent for which selective aptamers have been identified. As a perspective, this platform has the potential for the development of point-of-care detection avoiding to access the healthcare environment. The recent SARS-CoV-2 pandemic highlighted how this aspect would be critical in the management of such emergency conditions.

**Table 1:** Relevant *S. aureus* biosensors with their component properties and detection limit.

Biorecognition Element	Detection Method	Detection Limit	Ref.
PEI-GA modified antibody	amperometric	10 CFU/mL	[20]
lytic phage	surface plasmon resonance-based sensor	10^4^ CFU/mL	[21]
monoclonal antibody	optical fiber	10^4^ CFU/mL	[22]
hybridizing *S. aureus* DNA	electrochemical (multiwalled carbon nanotubes-chitosan-bismuth)	3.17 × 10^−14^ M	[24]
hybridizing *S. aureus* ssDNA	chitosan–Co_3_O_4_ nanorod–graphene	4.3 × 10^−13^ M	[58]
DNA aptamer	potentiometric (single-walled carbon nanotubes)	8 × 10^2^ CFU/mL	[10]
DNA aptamer	graphene interdigitated gold electrode	41 CFU/mL	[30]
biotynilated DNA aptamers	electrochemical (silver nanoparticles)	1.0 CFU/mL	[31]
DNA aptamer	fluorescence (labeled aptamer)	10^2^ CFU/mL	[32]
aptamer-conjugated gold nanoparticles	resonance light-scattering–detection system	single cells	[36]

## 2. Materials and Methods

### 2.1. In Silico Design

Two main criteria were followed during the aptamer design phases: (i) evaluation of the aminoacid-nucleotide pairing suitability according to the employed pseudo-code [49]; (ii) the selection, whenever possible, of residues bearing side chain with similar length with respect to the wild type ones.

### 2.2. Molecular Dynamics

The three-dimensional structure of SA23-2AII DNA single strand was generated using MC-Fold-MC-Sym pipeline [59]. Then, the protein-aptamer complex was obtained by manually placing the ssDNA molecules close to the CRO protein, according to the protein-DNA complex reported in PDB 6cro [60]. We reproduced the interactions hypothesized by the mentioned recognition code [49], residues 26–27 and 31–32 of CRO protein, which should interact with nucleobases at position 2–3 and 16–20 of SA23 DNA sequence. To optimize the molecules and the relative interaction, we submitted the SA23-2AII complex model to 500 ns of MD simulation using the GROMACS 5.0.5 package [61]. The complex was solvated in an octahedron box using the TIP3P water model, maintaining a 1.1 nm distance from the molecule border [62]. Counter-ions were added to neutralize the whole system. Temperature and pressure were controlled with the Berendsen algorithm [63], following previous protocols [64,65,66,67]. The Ewald method was applied to model electrostatic interactions. Before starting the equilibration, waters were minimized for 10 ps at 300 K, restraining protein and RNA atomic positions with a harmonic potential. Starting from 50 K, the system was heated up gradually to 300 K in six steps phases and the production run in NPT standard conditions for 500 ns, without restraints. The MD trajectory was analyzed with GROMACS, VMD [61,68] and Pymol [69] packages. The second part of the simulation was clustered to extract a representative conformation, with the gromos clustering method [70]. The structure having the lowest RMSD, with respect to the other cluster members, was considered as the most representative, for each cluster.

### 2.3. Peptide Synthesis and Labeling

Peptide synthesis was carried out on a microwave-assisted solid phase-based peptide synthesizer, followed by labeling with 5(6)-carboxyfluorescein [56,71].

### 2.4. Circular Dichroism

Circular dichroism (CD) spectra were recorded with a Jasco J-715 spectropolarimeter (Jasco International Co. Ltd., Tokyo, Japan) thermostatted with a Peltier unit set at 20 °C. Secondary structure estimation was performed by using the Dichroweb server [72,73].

### 2.5. Fluorescence Analysis

Fluorescence spectra of each peptide-aptamer complex were recorded on a Jasco FP-777 spectrofluorometer. The excitation wavelength was 488 nm and the emission wavelengths were in the range from 510 to 610 nm. Peptides tagged with carboxyfluorescein were dissolved in 20 μM phosphate buffer and titrated with different amounts of aptamer. The peptide-aptamer mixture was incubated at room temperature for 15 min to allow the formation of the complexes before the registration of each fluorescence spectrum.

### 2.6. Nanostructured Zirconia Substrates

Nanostructured zirconia (ns-ZrO_x_) thin films were grown on glass microscope slides by depositing supersonic beam seeded with zirconia clusters produced by a pulsed microplasma cluster source (PMCS) under high vacuum conditions [74,75,76]. A zirconium rod is ablated by a pulsed argon plasma stream, ignited by an electric discharge. The removed materials thermalize in the quenching inert gas and condense to form clusters. The mix of cluster and inert gas is extracted through a nozzle, forming the seeded supersonic beam. The zirconia clusters are collected on substrates mounted on the manipulator perpendicularly to the beam trajectory. Since the kinetic energy of the zirconia clusters is sufficiently low to avoid fragmentation a cluster assembled film is grown. The films are partially oxidized in the deposition apparatus due to the presence of oxygen traces; further oxidation occurs upon exposure to air.

The surface roughness of the substrates is crucial for the molecules immobilization since it affects their adsorption density and functionality [44,77,78]. Thus, the films were grown with deposition parameters optimized for producing films with morphological properties suitable for the aptamers immobilization. The rms roughness was estimated by AFM measurements, as extensively described elsewhere [75]. The value chosen as optimal was 15 nm.

### 2.7. Aptamers Microarrays

The validation and quantification of the aptamers immobilization on ns-ZrO_2_ have been achieved by using the protocol called protein surface interaction microarray (PSIM), which allows the high throughput study of biomolecules-surfaces interactions [43]. The protocol applied takes advantage of the biotin-streptavidin pairing to enhance the adsorption of aptamers on ns-ZrO_2._ Initially, a small volume droplet (450 pL) of Streptavidin is spotted on slides with the zirconia nanostructured surfaces, on top of those subsequent 450 pL droplets biotin-triethylene glycol (TEG) fluorescent aptamers are spotted. All the spotting process has been performed using an automated sciFLEXARRAYER S3-Scienion AG spotter (Scienion AG, Berlin, Germany). After incubation for 20 min at 65% controlled humidity, the slides were blocked in a solution with PBSMT (PBS + 5 mM MgCl_2_ + 0.1% (*v*/*v*) Tween 20) for 10 min and washed 3 times in PBSMT for 3 min, 3 times in PBSM (PBS + 5 mM MgCl_2_) for 3 min and finally in doubly-distilled H_2_O for 1 min. Slides were then dried under gentle nitrogen flux. The amount of adsorbed biomolecules is evaluated by reading the fluorescent signal with a TECAN microarray scanner (Tecan Group Ltd., Männedorf, Switzerland), and images are analyzed using Scan-array Express software (PerkinElmer Inc., Waltham, MA, USA). Figure S1 shows a sketch of PSIM protocol applied to 4 different fluorescent aptamers spotted on ns-ZrO_2_ substrate. SA23 has been purchased from Sigma-Aldrich (Sigma-Aldrich Corp., St. Louis, MO, USA), aptamer SA23 biotin-TEG 5′ modified has been purchased from PrimmBiotech (PrimmBiotech, Inc., Cambridge, MA, USA). The IIA2 fluorescent peptide is incubated in the aptamers microarray immediately after the blocking step. The concentration of the peptide is 20 µM in PBS and incubation time is of 15 min.

### 2.8. Fluorescence Confocal Microscopy

The microscope slides have been imaged in the xy and xz planes using a Leica TCS SP5 confocal microscope (Leica Microsystems, Wetzlar, Germany), using a diode (561 nm) laser with 30% laser power, a 40× oil immersion objective, 1024 × 1024 image resolution and UV (405 nm) at 35% laser power, 40× oil immersion objective and 1024 × 1024 image resolution (Figure S1).

### 2.9. Bacterial Culture

*Staphylococcus aureus subsp. aureus* (ATCC-25923) and *Escherichia coli* (ATCC-25922) are from LGC Standards (LGC Standards s.r.l., Milan, Italy). The bacteria are cultured in solution, using LB Broth (Miller) from Sigma-Aldrich as growth medium, in a thermoshaker at 37 °C overnight. Then the concentration of bacteria is measured using a spectrophotometer at fixed wavelength (600 nm). Before the incubation with the immobilized aptamers, the bacteria were stained with Hoechst 33,258 from Sigma-Aldrich (3 µL per mL of solution).

### 2.10. Detection of Bacteria-Aptamer Interaction

In order to detect the activity of aptamers immobilized on nanostructured surfaces for bacterial recognition the following protocol has been established: incubation with 5 µM streptavidin for 20 min; washing with PBSM 1 time for 5 min; incubation with 5 µM biotin-TEG-aptamer-Cy3 labelled for 15 min; washing with PBSMT 3 times for 3 min; incubation with *S. aureus* or *E. coli*, 10^8^ bacteria/mL for 45 min at RT; washing with PBSMT and PBSM, 3 times for 3 min each.

### 2.11. Bacterial Displacement

The bacterial displacement experiments are based on the previously described procedures: round cover glass (Ø 13 mm) coated with ns-ZrO_2_ (rms roughness 15 nm), coverage of the surface with streptavidin (6.63 µM, 100 µL), incubation at RT for 20 min in 65% controlled humidity, washing in PBSM, removal of PBSM, spotting of aptamer SA23 biotin-TEG modified (26.53 µM, 100 µL), incubation at RT for 20 min in 65% controlled humidity, washing in PBSM, removal of PBSM, spotting of peptide (50 µM, 100 µL), incubation at RT for 15 min in 65% controlled humidity (kept dark), plate reading (TECAN), washing in PBSM, bacteria plating incubation with *S. aureus* or *E. coli*, 10^8^ bacteria/mL for 45 min at RT (kept dark), washing with PBS, plate reading (Tecan) of the fluorescein signal (excitation wavelength 485 nm, emission wavelength 535 nm).

## 3. Results and Discussion

### 3.1. Aptamer Selection for In Silico Design of Interacting Peptide

**Figure 1:**
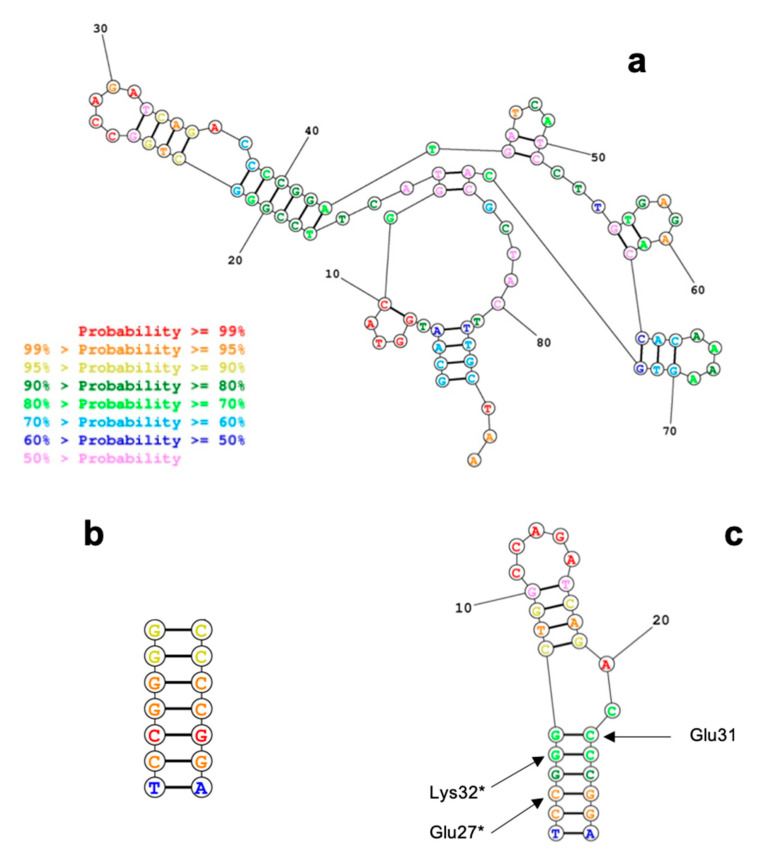
Aptamer folding prediction obtained with RNAstructure (https://rna.urmc.rochester.edu/RNAstructureWeb/) for (**a**). Full lenght SA23; (**b**). SA23 short1 sequence; (**c**). SA23 short 2. Here the predicted contacts with the mutated residues are also indicated. The color-code shows the level of prediction reliability, as indicated in the legend.

The five aptamers presented by Cao and coworkers [27] were considered for selecting the candidate that could be better targeted by peptides designed in silico. SA23 (Figure 1a) was chosen among these aptamers firstly for a structural peculiarity. According to the predicted secondary structure, in fact, it presents a relatively long trait in double strand configuration (the hairpin made by nucleotides 16–44), with high GC content in the inner portion. This double strand DNA region of the aptamer was then identified as the putative binding region of the peptides. Moreover, the SA23 aptamer results as the one with the higher affinity towards *S. aureus* within this group.

### 3.2. Peptide Scaffold Selection

Once the proper aptamer was chosen, we identified among proteins able to interact with nucleic acids λ-Cro as a suitable scaffold for the design of aptamer-interacting peptides [79]. λ-Cro is a 66 aa protein that plays a pivotal role in the switch from lysogenic to lytic phase in the growth cycle of phage λ and represented a suitable scaffold for several reasons: (i) the availability of three-dimensional structures both in the presence and absence of its cognate DNA (PDB ID: 6cro [60] and 5cro [80], respectively) allows detailed structural evaluations; (ii) the helix-turn-helix motif of the DNA binding domain is relatively small and all the interactions between the nucleobases of the consensus sequence and the peptidic backbone are well characterized; (iii) the minimum functional portion of the consensus sequence is quite short and made by contiguous nucleobases along the two strands of the DNA target; (iv) previous works reported successful examples of Cro reprogramming for binding consensus sequences that differ from the wild type [79]. Moreover, the helix-turn-helix peptide domain of the Cro protein showed to well mimic full-length protein for binding [79]. Therefore, the sequence of the helix-turn-helix motif of Cro was mutated, accordingly to the previously reported recognition code, to specifically bind SA23 double strand region.

### 3.3. In Silico Design and Synthesis of λ-Cro Mutant Specific for Aptamer Binding

The selected portion of λ-Cro sequence suitable for in silico evaluation and following mutation spans the residues 15–38 (wild type sequence: GQTKTAKDLGVYQSAINKAIHAG), that includes almost the totality of the a-specific and pseudo-specific residues that interact with its cognate DNA sequence, according to the complex reported by Olhendorf et al. (PDB ID: 6cro) [60]. The design was performed using the complex as template and considering the peptides acting as a monomer, which was reported to bind its operator site, with a binding constant of around 25 μM or above for the wild type. This binding constant is theoretically appropriate for a system where the labeled peptide has to be displaced by the aptamer that, on the contrary, shows binding constant in the nanomolar range.

The secondary structure of SA23 was predicted by using RNAstructure and is reported in Figure 1a. As mentioned, the paired region was chosen as possible target. The interacting peptides have been designed substituting the original residues to possibly generate more stable interactions with the paired aptamer (Table 2).

The peptides were synthesized using microwave assisted Fmoc-based solid phase peptide synthesis on Rinkamide resin [56]. Labelled peptides were prepared on resin using 5,6 carboxyfluorescein as the fluorescent tag [81].

### 3.4. λ-Cro Peptide Mutants Characterization by Far-UV Circular Dichroism

Circular dichroism experiments were carried out on λ-Cro peptide mutants optimized to interact with the double strand portion of SA23 aptamer in 20 mM phosphate buffer, pH 7.4. Circular dichroism was used to characterize the secondary structure of synthesized peptides, and hence to determine the mutation effect on the structure stability. It has already been demonstrated that it is possible to mimic the DNA binding behavior of λ-Cro by shorter, chemically synthesized peptides, representing the helix-turn-helix region of the protein [79]. These peptides, also in dimeric form, showed significant helical content only when α-amino isobutyric acid is introduced in the sequence [79]. To quantify the λ-Cro mutant helical content, the spectra of the peptides were analyzed using Dichroweb server (CDSSTR algorithm-reference set 7), yielding a low percentage of helical content (Table 3), highlighting that the isolated peptides in solution are unstructured in the absence of the target nucleotide sequences.

To verify that increasing the concentration the peptides does not result in the formation of aggregates or oligomeric forms, CD spectra were collected as a function of peptide concentration (10–100 µM). The CD spectra in this range of concentration appeared identical (data not shown). Peptide solutions demonstrated to be stable even after 1–2 freeze-thawing cycles.

The use of circular dichroism for evaluating the interaction between SA23 aptamer and the peptides requires also to characterize SA23 CD spectra. Since peptides were specifically designed to bind a specific region on the aptamer (the hairpin formed by nucleotides 16–44), shorter sequences were also characterized: SA23 short1 and SA23 short2, the former being the short paired sequence on which the peptide is expected to bind, while the latter is the complete hairpin. CD spectra of SA23, SA23 short1 (Figure 1b) and SA23 short2 (Figure 1c) were collected in the far-UV and near-UV regions.

### 3.5. Circular Dichroism Studies on Peptide-Aptamer Interaction

The peptide-aptamer interaction was first studied by near-UV CD spectroscopy. In this region, only aptamers and not peptides contribute to the spectra. The analysis of this spectral region allowed to monitor the peptide effect on the aptamer structure, without any interference from the peptide conformational change.

Solutions containing aptamers and peptides at the same concentration (20 µM) were incubated before recording the spectra. A comparison between the spectra of the aptamer-peptide mixture and the arithmetic sum of the two separated components would highlight potential interactions.

**Figure 2:**
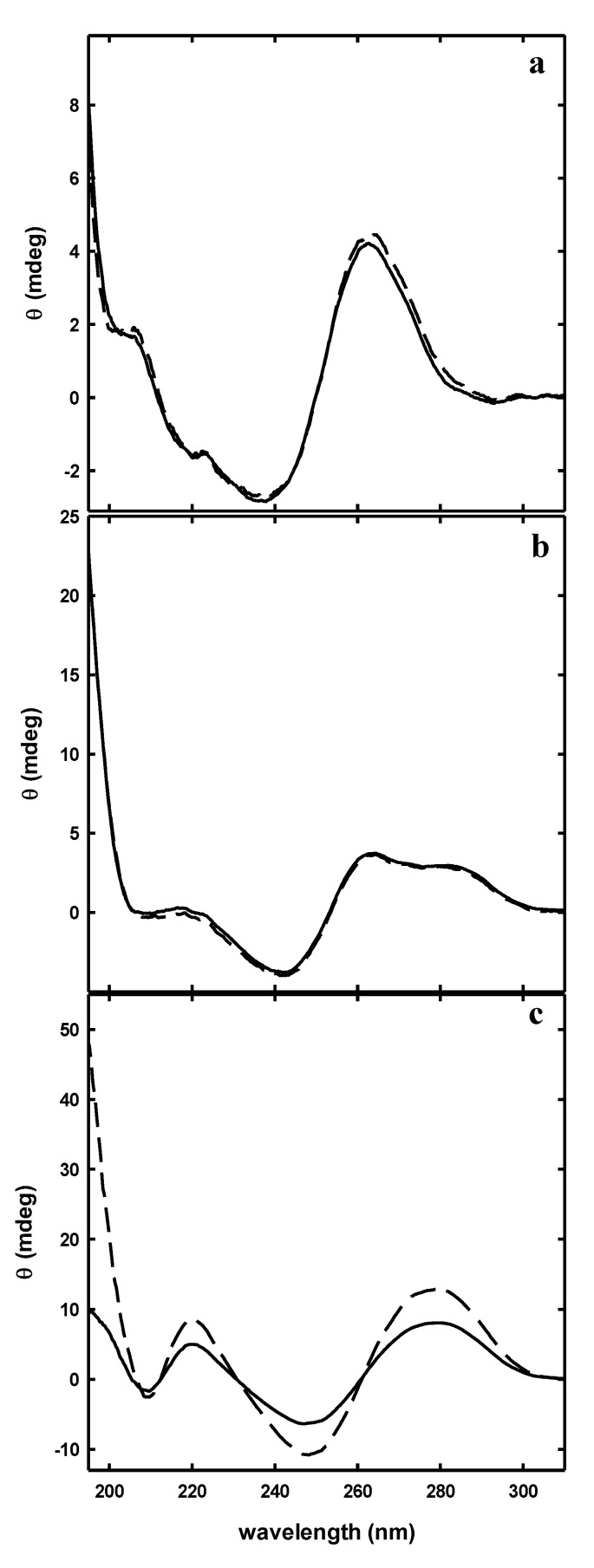
Circular dichroism spectra of the mixtures (solid lines) and the arithmetic sum (dashed lines) of peptide IIA2 with SA23 short1 (**a**), SA23 short2 (**b**) and SA23 (**c**).

The comparison between arithmetic and experimental mixtures highlighted no differences with the short versions of SA23, SA23 short1 and SA23 short2, while a marked difference was observed for the full length aptamer SA23 with the four peptides IIA2, IAser, IIA and IB2. In Figure 2 the comparison between arithmetic and experimental mixtures for the IIA2 peptide is shown. The absence of CD spectra difference between the mixtures and the arithmetic sums observed for the shorter versions of SA23 (SA23 short1 and SA23 short2) may derive from the lack of conformational adjustment upon peptide binding.

**Figure 3:**
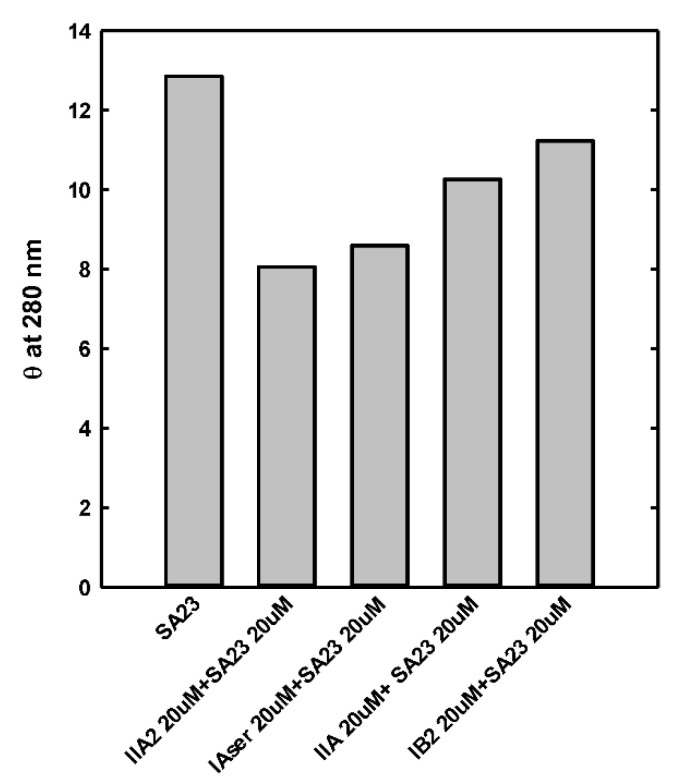
Circular dichroism signal at 280 nm of SA23 in the absence and presence of peptides.

However, the collected data showed that the aptamer SA23 interacts with four peptides, promoting a conformational change detectable by a CD band intensity decrease. The most evident effect was observed for peptide IIA2, as reported in Figure 3 where the ellipticity at 280 nm is reported.

To demonstrate that peptide binding is determined by the specific mutation inserted on λ-Cro peptide mutants, we synthesized and checked sequence-scrambled peptides, and peptides where mutations causing unfavorable interaction were inserted. The comparison of the mixture of these peptides with SA23 with the arithmetic sum gave overlapping results, demonstrating the absence of interaction (data not shown).

### 3.6. Fluorescence Characterization of Peptide-Aptamer Interactions

Based on CD results, further analyses were carried out by fluorescence spectroscopy, using the most promising IIA2 peptide labeled with fluorescein, in order to: (i) confirm peptide-aptamer complex formation also after peptide labeling, and (ii) calculate the dissociation constants (Kd) of the peptide-aptamer complex, which determination is prevented in circular dichroism measurements by experimental limits of acquiring CD spectra at low concentrations.

**Figure 4:**
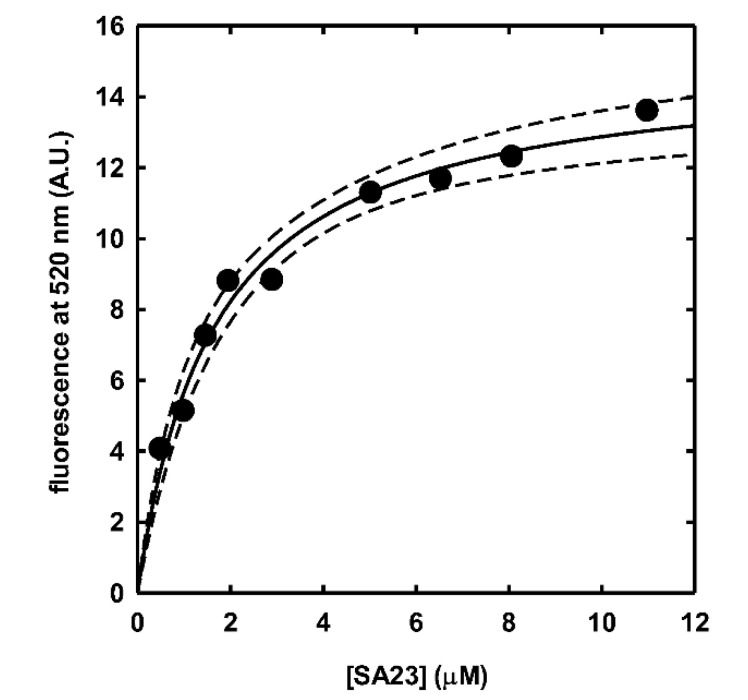
Binding of SA23 to fluorescein-labeled IIA2 peptide recorded by measuring fluorescence emission changes at 520 nm in 20 μM phosphate buffer at room temperature. Solid line, fitting of fluorescent data to a binding isotherm (K_d_ 1.64 ± 0.20 µM); dashed lines, 95% confidence bands.

The labeled peptide was titrated with increasing concentrations of aptamers and spectral perturbations were followed at 520 nm emission wavelength, allowing to calculate the dissociation constants (Figure 4). In accordance with the CD measurements, specific binding could be detected for the aptamer SA23 with peptide IIA2, with a measured K_d_ of 1.64 ± 0.20 µM. Differently form CD measurements, fluorescence analysis was able to detect peptide binding also to the shorter versions of SA23, SA23 short 1 and SA23 short 2, for which a K_d_ of 5.81 ± 0.21 and 9.61 ± 0.77 µM was obtained, respectively. These data demonstrated that IIA2 peptide is able to bind to SA23 with an affinity in a low micromolar range, and peptide binding is specific for the target sequence, as demonstrated by binding of SA23 short 1 and SA23 short 2, that showed similar binding affinity. IIA2 peptide affinity for SA23 aptamers, compared to the reported affinity of SA23 for *S. aureus* of 61.50 ± 22.43 nM, makes IIA2 peptide a good candidate for a device where the displacement of the peptide from SA23 binding site in the presence of the pathogen bacterium easily allows the *S. aureus* detection.

The fluorescence behavior of fluorescein upon binding will allow an easy detection of aptamer:peptide complex displacement by *S. aureus*. In fact, fluorescein loses fluorescence upon release from the aptamer, and this can be appreciated by the loss of fluorescence of the nanostructured chip upon its incubation in the putative contaminated fluid.

### 3.7. Molecular Dynamics Simulation

**Figure 5:**
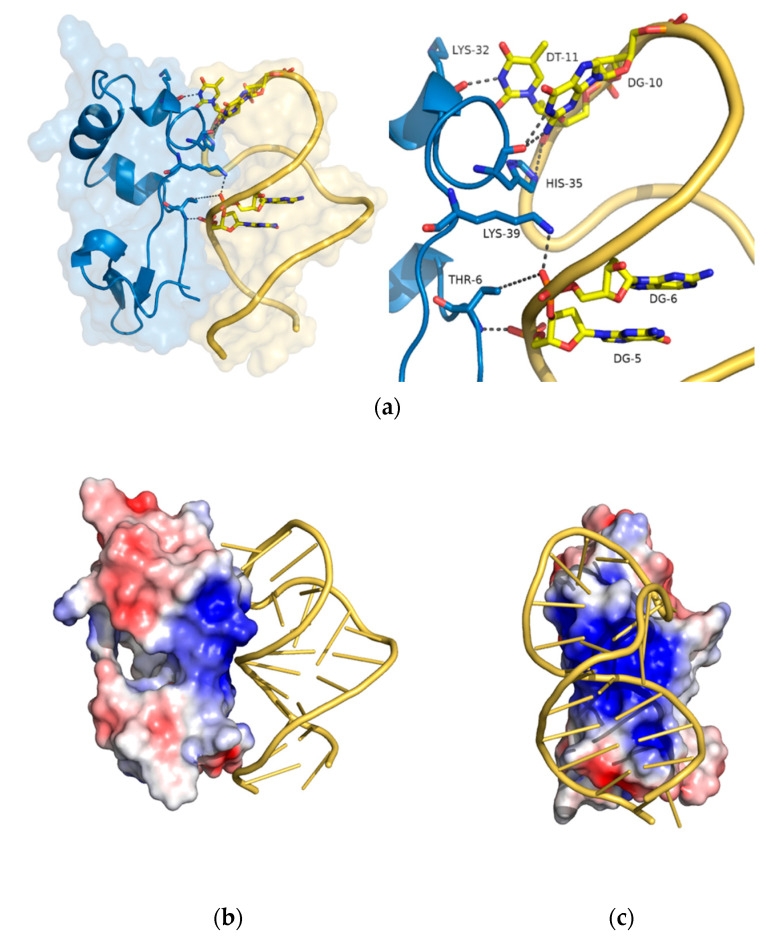
(**a**). Global (left) and zoom (right) view of the key interactions at the protein-aptamer interface. The protein is shown in blue cartoon and the aptamer in yellow ribbon; the atoms involved in each connection are labeled. (**b**,**c**). Frontal and lateral view of molecular dynamic representative structures of the protein-aptamer complex simulations derived using a RMSD based clustering approach. The CRO protein is shown as surface and colored according to the electrostatic potential. The red color (negative potential) arises from an excess of negative charges near the surface and the blue color (positive potential) occurs when the surface is positively charged (±1 kT/e). The white regions correspond to fairly neutral potentials. The aptamer is colored yellow.

To further investigate the interaction of SA23 with the most promising peptide IIA2, we modelled the aptamer-peptide complex, using as guide the coordinates of the CRO protein co-crystallized with a DNA duplex molecule (PDB ID 6cro [60], see method section for details). To stabilize and investigate the dynamic and structural properties of the complex, molecular dynamic simulation (MD) in explicit waters was performed for 500 ns. Although no major perturbations affect the complex, the root mean square deviation (RMSD) values exhibited by either the complex, the peptide or the aptamer, with respect to the initial state, reveal the system reaches a global equilibrium in the second half of MD simulation (Figure S2). In Figure 5 (panel a) a representative structure of the equilibrated part of the trajectory is shown. Even if changes occurred with respect to the starting modelled structure, a good protein-aptamer interface is established and preserved along the trajectory and the aptamer maintains the same loop arrangement dictated by the original folding. We have calculated the occurrence percentage of hydrogen bonds formed at the complex interface, finding that 3 out of the 7 significative hydrogen bonds (preserved for more than 30% of the trajectory frames) involve the aptamer backbone atoms (Figure S3 and Table S1). The remaining connections involve nucleobase atoms of the aptamer. In detail, Lys39 and Thr6 (out from the peptide length) interact with the phosphate groups of Guanine 5 and 6 (Table 2), Lys32 and His35 make persistent bonds with the nucleobases of guanine 10 (DG10) and timine 11 (DT11). The last interactions are formed by the exposure of Lys32 and His35 out of the double strain towards the peptide and are able to anchor the peptide with specific nucleobase interactions. Likely, the IIA2 peptide sequence is able to maintain the required secondary structure arrangement to bind the aptamer through non-specific and specific contacts.

The typical electrostatic profile of the protein-aptamer interface, showing positively charged patches on the peptide side in contact with the negatively charged nucleic acid backbone, is shown in Figure 5 (panel b and c).

### 3.8. Aptamer Immobilization on Ns-ZrO_2_

**Figure 6:**
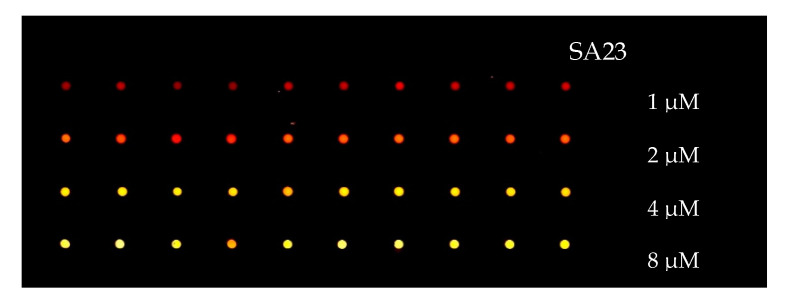
Scanned image of aptamer-microarray. SA23 spotted on ns-ZrO_2_ surface covering a glass slide with different concentrations of 1, 2, 4 and 8 µM.

To exploit the possibility to realize an aptamer-based biosensor for bacteria detection the microarray technique was applied to test different aptamers and different experimental conditions for their immobilization in one single experiment and to test bacteria-aptamer interactions. Figure 6 shows the image acquired by a scanner/reader of SA23 fluorescent aptamer spotted on a glass slide coated with ns-ZrO_2_ for various concentrations, namely 1, 2, 4 and 8 µM.

**Figure 7:**
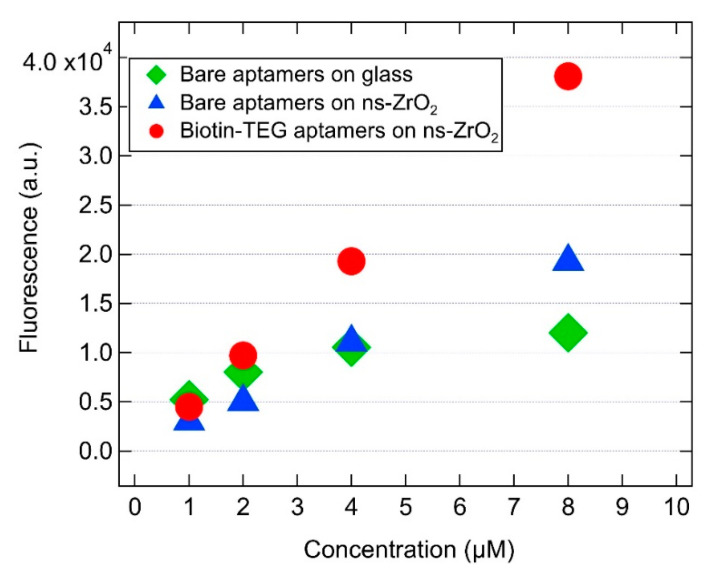
Isotherm describing the adsorption behavior of bare aptamers spotted on glass and on ns-ZrO_2_ and of aptamers functionalized with biotin-TEG 5′ spotted on ns-ZrO_2_ coated with streptavidin.

The isotherms showing the adhesion of SA23 aptamer on different substrates are reported in Figure 7. The promotion of the adhesion of bare aptamers on ns-ZrO_2_ substrate with respect to clean glass is mainly evident at high concentrations. However, the adhesion enhancement is of about a factor of two for all the concentrations, when the biotinylated aptamers pair with streptavidin deposited on the nanostructured surface. Furthermore, no manifest hindering to aptamer adhesion appears to be induced by the TEG spacers.

### 3.9. Aptamers Hybridization with Peptides

**Figure 8:**
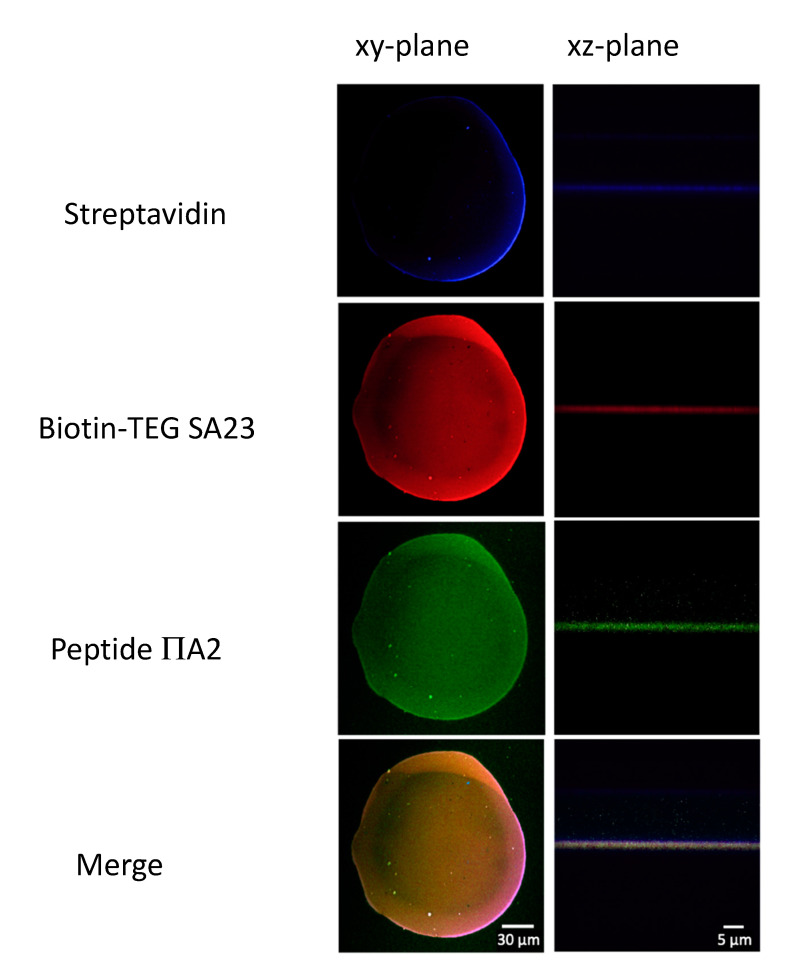
Confocal microscopy images in the focal plane and along the *z*-axis. From top to bottom: spot of streptavidin physico-absorbed on cluster-assembled zirconia surface, SA23 aptamer functionalized with biotin-TEG immobilized on the surface via biotin-streptavidin bindings, peptide used as probe for the SA23 aptamer and merge of the three fluorescence signals.

The SA23 aptamer immobilized on the microarray was left to hybridize with fluorescein-labeled IIA2 peptide. The total absence of fluorescein signal suggests that aptamers loose their functionality, possibly because of interactions with the microarray surface leading to incorrect aptamer folding, i.e., electrostatic interactions between the negatively charged DNA phosphodiester backbone and the positively charged surface. To overcome this problem, biotin-TEG modified aptamers were used since the 15 atoms spacer should be able to avoid any possible interaction between the aptamers and the surface [82]. Thus, a new microarray with biotin-TEG aptamers spotted on ns-ZrO_2_ coated with streptavidin was left to hybridize with the fluorescein-labelled peptide (IIA2). The assays result is reported in Figure 8 and Figure S4. The expected signal originating from the hybridization between SA23 and IIA2 was clearly appreciable, demonstrating that the SA23 aptamer was immobilized with the correct folding.

### 3.10. Interaction of S. aureus with SA23 Aptamer

**Figure 9:**
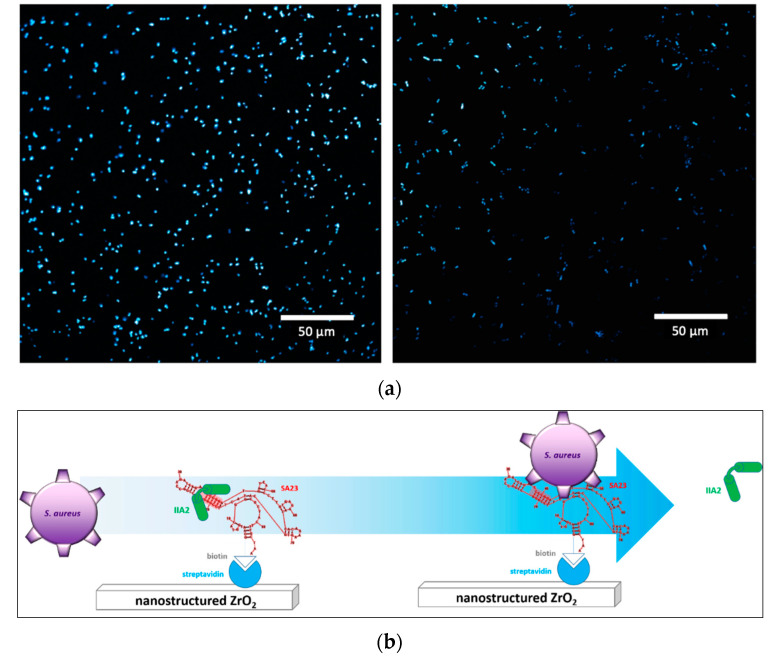
(**a**). Confocal microscope images of bacteria stained with Hoechst, upon interaction with previously immobilized SA23 aptamer. Left: *S. aureus*; right: *E. coli*. Both bacteria have been incubated at a concentration of 10^8^ bacteria/mL. (**b**). schematic representation of the device assembly.

Figure 9 shows representative confocal images from multiple acquisitions of the interaction of *S. aureus* (left panel a) and *E. coli* (right panel a) with SA23 aptamers immobilized on ns-ZrO_2_. The larger amount of fluorescent spots for *S. aureus* in comparison with those for *E. coli* indicates a high degree of hybridization of the former bacteria with the aptamer, supporting the effective selectivity of the device. This achievement is a fundamental step for the further device assessment, i.e., the peptide displacement (Figure 9, panel b).

### 3.11. Aptamer:Fluorescent Peptide Complex Displacement by S. aureus

**Figure 10:**
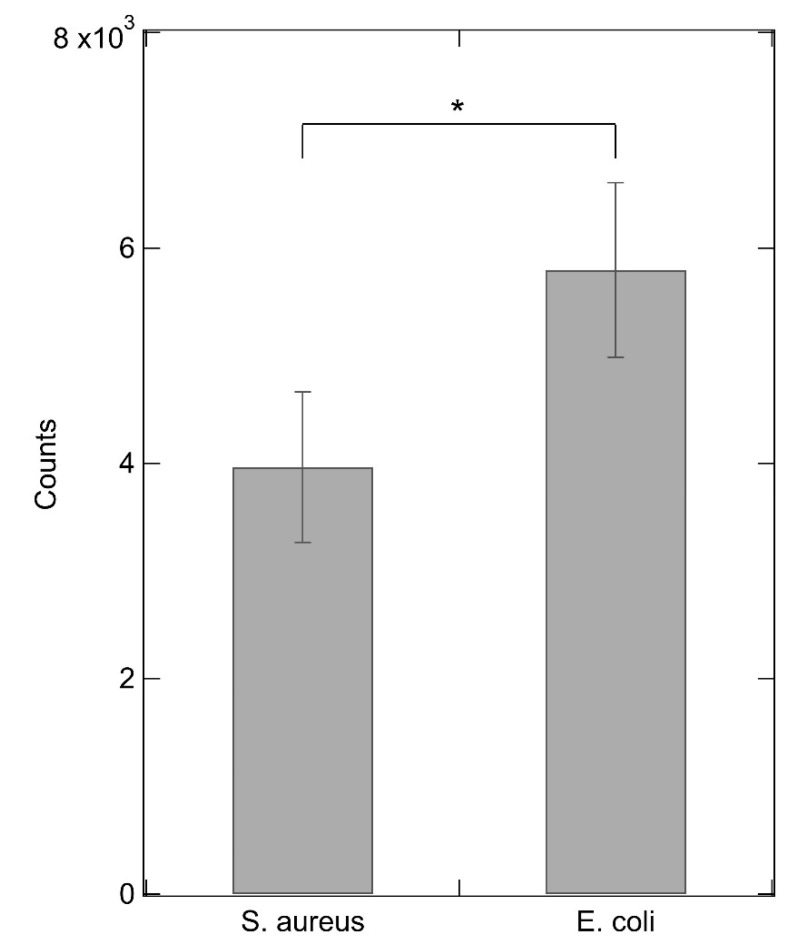
Fluorescence counts of fluorescein-labeled IIA2 peptide upon incubation and washing with *S. aureus* and *E. coli*. All data were obtained from two independent experiments (n = 15). Error bars represent the standard deviation (* *p* < 0.05, unpaired t test).

To finally test the functionality of the proposed device, we investigated the capability of *S. aureus* to outcompete IIA2 peptide binding to SA23. The ns-ZrO_2_ substrate functionalized with SA23 and hybridized with IIA2 peptide was subjected to bacterial incubation followed by a washing step; then fluorescence was measured. Successful competition of bacteria with respect to the fluorescently labelled peptides for binding to SA23 is expected to bring about fluorescence loss upon washing. We compared the fluorescent peptide displacement by *S. aureus* and *E. coli* to evaluate the selectivity of peptide displacement by SA23-*S. aureus* interaction. The residual fluorescence signal, averaged on 16 samples using a plate reader, is related to the remaining amount of bound peptides, that is inversely related to their displacement by bacteria. The data shown in Figure 9 (panel a) and analyzed in Figure 10 indicate that the device plated with *S. aureus* exhibits a significantly lower signal with respect to that with *E. coli.* This result supports the effectiveness of the device for selective detection of *S. aureus.*

## 4. Conclusions

We demonstrated that the rational design of a peptide able to interact with a nucleotide sequence (aptamer) selected for the recognition of a specific pathogen is a viable approach for the development of a biosensor. Moreover, we show that the developed zirconia nanostructured substrate is a promising platform for generating biosensors based on the immobilization of receptors for the detection of pathogenic agents.

Important structural determinants related to the aptamer sequence and responsible for the specificity of the protein targeting have been revealed. MD simulations performed on the modelled peptide-aptamer complex validated the interaction and identified key residues fundamental for the complex stabilization. Even if the original interaction pattern, as predicted by the protein-DNA recognition code [49], has not been totally preserved, these results are of significant relevance, considering that no X-ray structure of the peptide-aptamer is available and the complex has been hardly modelled on the structure of the λ-Cro protein interacting with a double strand DNA. This likely supports the use of the recognition code for the prediction of key residue-nucleobase contacts in protein-nucleic acids interaction.

The microarray technique allows the development of biosensors to screen in parallel for more targets, given the possibility to immobilize multiple receptors on the same substrate, retaining their structure and functionality. Moreover, the high surface-to-volume ratio that characterizes the porous materials used as substrates for the microarrays, allows the adsorption of a higher amount of molecules/receptors/aptamers with respect to a flat surface.

The cluster-assembled zirconia developed in this work was used as reliable support for bioactive molecules’ immobilization, particularly aptamers, for biosensing applications. The protocol developed for their immobilization preserves the functionality of the nucleotides. This immobilization technique well couples with the use of fluorescently labeled synthesized peptides able to selectively bind specific aptamers.

We finally created a device where the fluorescent chip, loaded with the labeled peptide, looses fluorescence upon bacterium displacement and consequent peptide release in the fluid. A colorimetric switch as a detected signal could also be possible by preparing peptides labeled with solvatochromic fluorophores [83,84].

This device development platform can, in principle, be applied to any analyte for which selective aptamers have been identified, and whose rapid and specific point-of-care detection is desired.

## Figures and Tables

**Table 2:** λ-Cro mutant peptides sequence.

Peptide	Sequence	MW (Da)
IA	Ac-GQTKTAKDLGVYKSAIEEAIHAG	2428.73
IAser	Ac-GQTKTAKDLGVYKDAIEEAIHAG	2456.73
IB2A	Ac-GQTKTAKDLGVYDSAIEEAIHAG	2415.63
IIA	Ac-GQTKTAKDLGVYESAIEEAIHAG	2429.67
IIA2	Ac-GQTKTAKDLGVYEDAIEKAIHAG	2456.74
IIA3	Ac-GQTKTAKDLGVYEDAIEFAIHAG	2475.74
IIA2M	Carboxyfluorescein-GQTKTAKDLGVYEDAIEKAIHAG	2773
IA3M	Carboxyfluorescein- GQTKTAKDLGVYEDAIEFAIHAG	2792

**Table 3:** Peptides secondary structure estimated by Dichroweb server.

Peptide	Helices	Strand	Turns	Disordered
IA	17%	28%	18%	36%
IAser	21%	24%	20%	35%
IB2	21%	26%	21%	32%
IIA	19%	24%	20%	37%
IIA2	7%	32%	23%	37%
IIA3	5%	36%	17%	40%

## Supplementary Materials

The following materials are available online at https://www.mdpi.com/1424-8220/20/17/4977/s1: Peptide synthesis and labeling, Figure S1: Sketch of PSIM protocol applied to one fluorescent aptamer spotted on ns-ZrO_2_ at different concentrations. Upper left: spotting; upper right: incubation in a controlled atmosphere (65% humidity), immersion in a blocking solution and rinsing; bottom left: drying and bottom right: scanned image, Figure S2: Time evolution of the RMSD values with respect to the starting model. The RMSD values have been computed considering the C alpha and C5’ atoms of the protein and aptamer, respectively. The following color code was used: overall complex (mean: 0.7 nm, SD: 0.12 nm): black line, peptide 2AII (mean: 0.45 nm, SD: 0.07 nm): red, aptamer (mean: 0.59 nm, SD: 0.14 nm): green, Figure S3: Absolute number of protein-aptamer interfaces hydrogen bonds during the entire trajectory, Figure S4: Sketch of the biotin-streptavidin paring strategy. The aptamers are functionalized with biotin-TEG 5′and the ns-ZrO2 is coated with streptavidin. The 15-atom tetraethylene glycol (TEG) spacer is added for minimizing steric hindrance when conjugating the biotin with other molecules, Table S1: Persistent hydrogen bonds computed for the last 250 ns of simulation time.

## Abbreviations

The following abbreviations (in order of appearance) are used in this manuscript:

PCR	polymerase chain reaction
PNA	peptide nucleic acid
MALDI-TOF	matrix assisted laser desorption ionization time-of-flight
MS	mass spectrometry
SELEX	systematic evolution of ligands by exponential enrichment
CNT	carbon nanotubes
SARS-CoV-2	severe acute respiratory syndrome coronavirus 2
PEI-GA	glutaraldehyde pre-coated with polyethyleneimine
CFU	colony forming unit
PDB	protein data bank
MD	molecular dynamics
CD	circular dirchoism
ns-ZrOx	nanostructured zirconia
PMCS	pulsed microplasma cluster source
AFM	atomic force microscopy
PSIM	protein surface interaction microarray
TEG	triethylene glycol
PBS	phosphate buffed saline
RT	room temperature
RMSD	root mean square deviation
