# Supplementary material for: Rational Design of a User-Friendly Aptamer/Peptide-Based Device for the Detection of Staphylococcus aureus
Source: Sensors (Basel). 2020 Sep 2;20(17):4977. doi: 10.3390/s20174977 (PMC7506613; doi:10.3390/s20174977)
Supplement: Supplementary file 1 [file sensors-20-04977-s001.pdf]

Rational Design of a User-friendly Aptamer/Peptide-Based Device for the Detection of *Staphylococcus Aureus*

Luca Ronda [1,2], Alessandro Tonelli [3], Elisa Sogne [4], Ida Autiero [5,6], Francesca Spyrakis [7], Sara Pellegrino [8], Giorgio Abbiati [8], Elisa Maffioli [9], Carsten Schulte [4], Riccardo Piano [1], Pietro Cozzini [10], Andrea Mozzarelli [2,10], Stefano Bettati [1,2,*], Francesca Clerici [8], Paolo Milani [4], Cristina Lenardi [4], Gabriella Tedeschi [9], Maria Luisa Gelmi [8].

1 Department of Medicine and Surgery, University of Parma, Parma 43125, Italy; luca.ronda@unipr.it (L.R.); riccardo.piano@studenti.unipr.it (R.P.)
2 Institute of Biophysics, National Research Council, Pisa 56124, Italy; andrea.mozzarelli@unipr.it
3 DNAPhone s.r.l., Parma 43126, Italy; alessandro.tonelli @dnaphone.it
4 CIMAINA and Department of Physics, University of Milan, Milan 20133, Italy; eli.sogne@gmail.com (E.S.); carsten.schulte@unimi.it, (C.S.); paolo.milani@mi.infn.it (P.M.); cristina.lenardi@mi.infn.it (C.L.)
5 Molecular Horizon s.r.l, Perugia 06084, Italy; ida@moldiscovery.com
6 Institute of Biostructures and Bioimaging, National Research Council, Naples 80145, Italy
7 Department of Drug Science and Technology, University of Turin, Turin 10125, Italy; francesca.spyrakis@unito.it
8 Department of Pharmaceutical Sciences, University of Milan, Milan 20133, Italy; sara.pellegrino@unimi.it (S.P.); giorgio.abbiati@unimi.it (G.A.); francesca.clerici@unimi.it (F.C.); marialuisa.gelmi@unimi.it (M.L.G.)
9 CIMAINA and Department of Veterinary Medicine, University of Milan, Milan 20133, Italy; elisa.maffioli@unimi.it (E.M.); gabriella.tedeschi@unimi.it (G.T.)
10 Department of Food and Drug, University of Parma, Parma 43124, Italy; pietro.cozzini@unipr.it
* Correspondence: stefano.bettati@unipr.it; Tel.: +39-0521-905502

Peptide Synthesis and Labeling

Fmoc Rinkamide resin, Fmoc- amino acids, coupling reagents and DIEA were purchased from Zentek (Italy). Solvents and all other reagents were purchased from Sigma-Aldrich (Germany). All peptides were produced by microwave assisted solid phase peptide synthesis based on Fmoc chemistry on a CEM Liberty peptide synthesizer. They were purified using RP-HPLC with a Jasco BS-997-01 instrument and a DENALI C-18 column from GRACEVYDAC (10 µm, 250 x 22 mm). ESI mass spectra experiments were performed on a LCQ Advantage spectrometer from Thermo Finnigan (San Jose, CA, USA). All peptides were synthesized on a Rink amide MBHA resin (0.35 mmol/g) using a five-fold excess of Fmoc-amino acids (0.2 M in NMP), HOBT/HBTU (5 eq, 0.45 M 190 in DMF) as activators, DIEA (10 eq, 1 M in NMP) as a base. The couplings were performed with 5 min microwave irradiation at 75 °C (50 °C in the case of histidine in order to avoid racemization). Piperidine (20% in DMF) with 3 min microwave irradiation at 75 °C was used for Fmoc deprotection. The labelling was performed using 10 eq of 5(6)-carboxyfluorescein and HOBT/DIC), in the dark under vigorous shaking for 1 hour. 20% piperidine in dimethylformamide was then added shaking for 1 hour.

Reagent K (TFA/phenol/water/thioanisole/EDT; 194 82.5/5/5/5/2.5) for 180 min was used for the cleavage and then peptides were precipitated using ice-cold ethyl ether. All peptides were purified by RP-HPLC with a gradient elution of 5–70% solvent B (solvent A: water/acetonitrile/TFA 95/5/0.1; solvent B: water/acetonitrile/TFA 5/95/0.1) over 20 min at a flow rate of 20 mL/min. They were freeze-dried and stored at 0 °C.

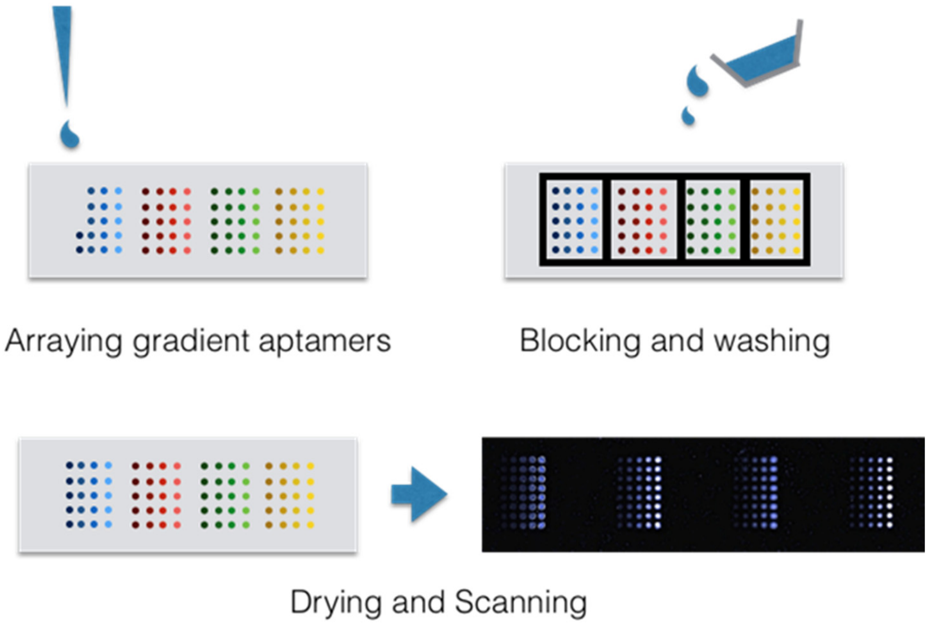

Figure S1. Sketch of PSIM protocol applied to one fluorescent aptamer spotted on ns-ZrO_2 at different concentrations. Upper left: spotting; upper right: incubation in a controlled atmosphere (65% humidity), immersion in a blocking solution and rinsing; bottom left: drying and bottom right: scanned image.

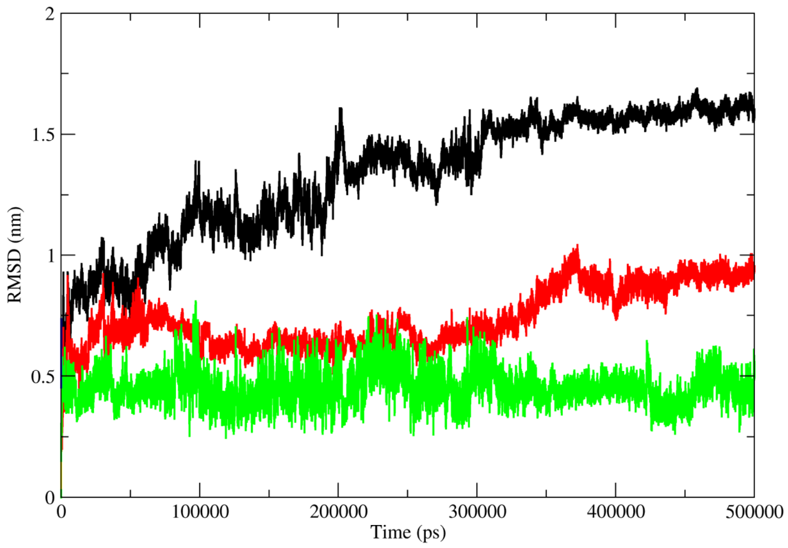

Figure S2. Time evolution of the RMSD values with respect to the starting model. The RMSD values have been computed considering the C alpha and C5' atoms of the protein and aptamer, respectively. The following color code was used: overall complex (mean: 0.7 nm, SD: 0.12 nm): black line, peptide 2AII (mean: 0.45 nm, SD: 0.07 nm): red, aptamer (mean: 0.59 nm, SD: 0.14 nm): green.

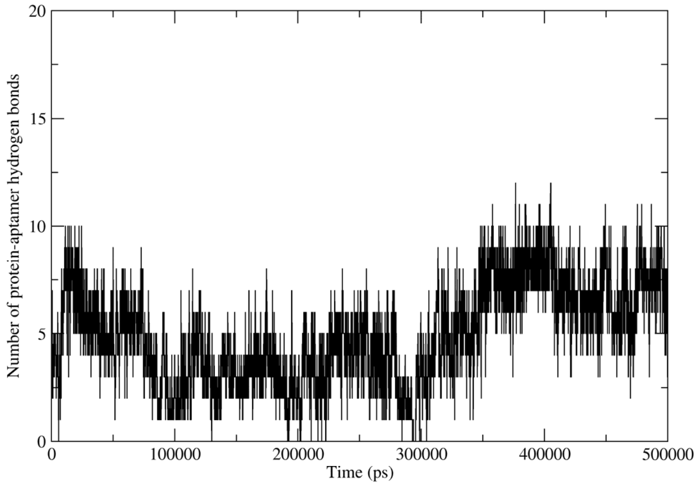

Figure S3. Absolute number of protein-aptamer interfaces hydrogen bonds during the entire trajectory.

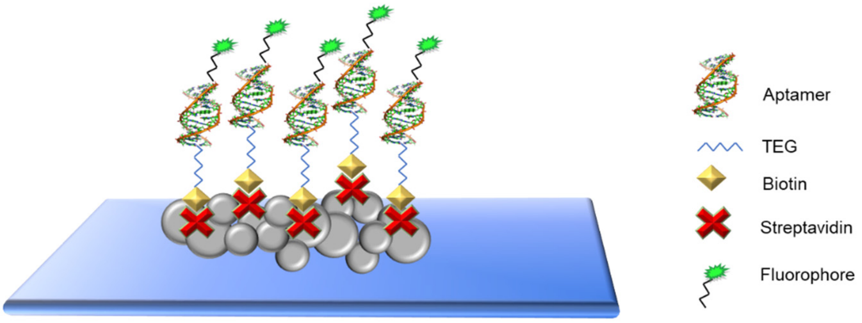

Figure S4. Sketch of the biotin-streptavidin paring strategy. The aptamers are functionalized with biotin-TEG 5′and the ns-ZrO_2 is coated with streptavidin. The 15-atom tetraethylene glycol (TEG) spacer is added for minimizing steric hindrance when conjugating the biotin with other molecules.

Table S1. Persistent hydrogen bonds computed for the last 250 ns of simulation time.

Protein Atom	Aptamer Atom	Occurrence (%)
THR6 (N)	DG5 (O2P)	53
HIS35 (O)	DG10 (N2)	52
LYS32 (O)	DT11 (N3)	51
THR6 (OG1)	DG5 (O2P)	41
LYS39 (NZ)	DG6 (O1P)	33
HIS35 (NE2)	DT11 (O3’)	31
HIS35 (O)	DG10 (N1)	30
